# DNA Metabarcoding Reveals Unexpected Predator–Prey–Microbial Dynamics in the Southern Right Whale (*Eubalaena australis*)

**DOI:** 10.1111/mec.70442

**Published:** 2026-06-15

**Authors:** Aashi Parikh, Richard O'Rorke, Emma L. Carroll, Els Vermeulen, Robert Harcourt, Stephanie Plön, William J. Rayment, Anthony Chariton

**Affiliations:** ^1^ School of Natural Sciences Macquarie University Macquarie Park New South Wales Australia; ^2^ School of Biological Sciences Waipapa Taumata Rau – University of Auckland Auckland New Zealand; ^3^ Mammal Research Institute Whale Unit, Faculty of Natural and Agricultural Sciences University of Pretoria Hatfield South Africa; ^4^ BioConsult SH Husum Germany; ^5^ Department of Marine Science Ōtākou Whakaihu Waka – University of Otago Dunedin New Zealand

**Keywords:** diet, eDNA, gut microbiome, krill, migration

## Abstract

Southern right whale ( *Eubalaena australis* ; SRW) populations are recovering from the impacts of commercial whaling, however, recovery has been spatially variable, with strong associations between reproduction and prey availability. The diet of SRWs has not been widely examined, and with SRW foraging shifting away from high‐latitude foraging grounds dominated by krill, it is essential to understand their diet at different locations. The gut microbiome is closely linked to diet, and characterising gut bacterial composition can help evaluate long‐term changes in prey and ecosystem dynamics. We used DNA metabarcoding to characterise the diet and faecal microbiome of SRWs from three calving/socialising grounds and a low‐latitude foraging ground. SRW feeding was more opportunistic than previously documented. Decapoda emerged as a key component of the SRW diet, being consistently detected at higher frequency and relative read abundance than euphausiids and copepods in whales from both calving/socialising and foraging grounds. The expected prey of Calanoida were also prominent in whales from the foraging ground, as were Stomatopoda, Cumacea and Semaeostomeae. A significant correlation between diet composition and faecal bacterial composition was observed, with euphausiids being the strongest predictor of bacterial variation. Our findings provide a new understanding of the breadth and diversity of the diet of SRWs. We also provide a baseline for monitoring diet–gut microbiome interactions. Collectively, these results offer a glimpse into the trophic dynamics of a Southern Ocean predator being impacted by climate‐driven changes in zooplankton distribution, with implications for long‐term population recovery.

## 1 Introduction

The Southern Ocean ecosystem is subject to warming temperatures, reductions in pH and intensifying climatic events (Gutt et al. 2015). This has significantly impacted the distribution of keystone prey, such as Antarctic krill ( *Euphausia superba* ), which are sensitive to changes in sea surface temperature (SST) and sea ice extent (Yang et al. 2021). As a result, Southern Ocean predators have faced nutritional deficits, with cascading effects on their health and reproduction (Agrelo et al. 2021; Vermeulen et al. 2023). The southern right whale (*Eubalaena australis*; SRW) is a large baleen whale which undertakes seasonal migrations between high‐latitude, productive summer foraging grounds and mid‐ to low‐latitude winter calving/socialising grounds, which tend to be resource‐poor (Harcourt et al. 2019). SRWs were intensely exploited during the commercial whaling period, with an estimated ~120,000 whales found pre‐whaling across 12 wintering grounds, reduced to 300–400 whales by around 1920 (Jackson et al. 2008; Harcourt et al. 2019). Since the end of illegal Soviet whaling in the 1970s, recovery has been spatially variable, with an estimated global population of 12,000 to 15,000 SRWs recorded in 2009 (Harcourt et al. 2019). For example, recovery at winter calving/socialising grounds around mainland New Zealand and eastern Australia has been much slower in comparison to New Zealand's sub‐Antarctic Maungahuka Auckland Islands (hereafter Auckland Islands) and south‐western Australia (Carroll et al. 2011, 2015).

To date, the diet of SRWs has been investigated via stomach content analysis, visual observation, stable isotope analysis, faecal prey remains analysis, zooplankton sampling and video‐imaging tags (Tormosov et al. 1998; Hoffmeyer et al. 2010; D'Agostino et al. 2016, 2023, 2024; Valenzuela et al. 2018). Stomach content analysis from Soviet whaling records (1951–1971) found that SRW diet was predominantly euphausiids (krill) south of 50° S, copepods north of 40° S, and a mix of both in between (Tormosov et al. 1998). More recent observations suggest a more complex picture, with whales feeding on squat lobster larvae (*Grimothea gregaria*) around the Auckland Islands, New Zealand, ~50° S (Carroll unpublished data), squat lobster larvae and copepods off Patagonia, South America between ~40°–50° S (Matthews 1938), and zooplankton patches dominated by copepods and euphausiids, but also including ctenophores, cladocerans and decapods at Peninsula Valdés, Argentina (D'Agostino et al. 2023, 2024). It has been suggested that SRWs and right whales in general may opportunistically feed on any densely congregated prey of the right size to be filtered through their baleen and which are slow to escape (Kenney 2009).

Historically, it was assumed that SRW feeding occurred primarily at high to mid‐latitude summer foraging grounds and that the whales fasted during the winter calving/socialising period at lower latitudes (Tormosov et al. 1998). However, it is now established that feeding may occur in all seasons (Torres et al. 2017; D'Agostino et al. 2024; Kennedy et al. 2024). For example, the Benguela Large Marine Upwelling System (15° S—34° S) on the South African west coast hosts low‐latitude foraging grounds such as St Helena Bay where whales have been observed feeding and socialising year‐round, with numbers peaking in spring and summer (Barendse and Best 2014).

Changes in krill distribution at high‐latitude foraging grounds have been linked to a decline in the health of several SRW populations (Agrelo et al. 2021; Grundlehner et al. 2025; Vermeulen et al. 2025). SRWs from Brazil and Argentina show declining reproductive rates linked to the abundance of krill at foraging grounds off South Georgia (~55° S) under the influence of the El Niño Southern Oscillation (ENSO; Agrelo et al. 2021). SRWs in South Africa have shown a 23% reduction in maternal body condition from the 1990s to the 2010s, alongside a significant increase in calving interval from 3 years to 4–5 years, attributed to changes in foraging success and prey distribution (Vermeulen et al. 2023, 2025; Germishuizen et al. 2024). Declining reproductive rates have also been observed in the south‐western Australian SRW population, with an observed increase in inter‐calf‐interval from 3.2 years (1996–2016) to 4.0 years after 2016 (Charlton et al. 2022). Mid‐latitude SRW foraging grounds have maintained their stability across four centuries in comparison to high‐latitudes (Derville et al. 2022), with satellite tracking demonstrating that high proportions of New Zealand and Australian SRWs prefer feeding at the subtropical convergence located at ~40° S (Riekkola et al. 2025). Despite this, the recovery of these populations has also begun to decline in the last decade (Grundlehner et al. 2025). Given SRW recovery varies greatly across regions and is associated with prey availability and composition, characterising the diet of SRWs in different foraging grounds and understanding the importance of supplementary feeding on calving/socialising grounds is key to understanding how whales might adapt to changing prey availability.

In this study, we sampled SRWs at three winter calving/socialising grounds—the Auckland Islands, subantarctic New Zealand; Fowlers Bay, Australia; and Algoa Bay, southeast coast South Africa—and a low‐latitude foraging ground—St Helena Bay, west coast South Africa. Zooplankton communities across these four coastal systems reflect the influence of local oceanographic forces. The waters surrounding the Auckland Islands support a low‐biomass community capable of efficient trophic transfer, structured by iron‐limited primary production and frontal oceanography, and dominated by copepods ( *Neocalanus tonsus* , *Calanus simillimus* , *Oithona similis* ), euphausiids (*Thysanoessa* spp.), salps, pteropods and chaetognaths (Bradford‐Grieve et al. 2003; Halfter et al. 2022). In the eastern Great Australian Bight, encompassing Fowlers Bay, a mesotrophic copepod‐dominated community is spatially and temporally structured by the Bonney Upwelling, which drives seasonal biomass and supports the highest sardine and anchovy densities in Australian waters (van Ruth and Ward 2009; Kämpf et al. 2023). In Algoa Bay, episodic upwelling and riverine input drive a seasonally influenced warm‐temperate assemblage dominated by copepod species and meroplankton alongside pelagic fish larvae (Dali 2011; Costalago et al. 2018). St. Helena Bay, which lies within the southern Benguela upwelling system, supports one of the region's most productive zooplankton communities, historically dominated by large calanoid copepods ( *Calanoides carinatus* , *Calanus australis*, *Centropages brachiatus*) and euphausiids ( *Euphausia lucens* ), though long‐term shifts towards smaller‐bodied species have been documented since the mid‐twentieth century (Gibbons and Hutchings 1996; Gibbons et al. 1999; Huggett et al. 2009). Across all four systems, copepods appear to constitute the dominant zooplankton group.

The gut microbiome is closely associated with diet and the digestion of prey, and can significantly influence diet specialisation in mammals (Ley et al. 2008). In bowhead whales ( *Balaena mysticetus* ), a close relative of SRWs, the digestion of wax esters, the primary lipid in krill, is strongly associated with the gut microbiome and specific bacterial taxa (Miller et al. 2020). Gut bacteria in mammals are frequently responsible for digesting food components that otherwise could not be broken down by the digestive system (Miller et al. 2020). Characterising the gut microbiome alongside prey composition thus allows for a more comprehensive view of the digestive potential of an animal and may provide insights into dietary patterns (Choi et al. 2022). Additionally, the gut microbiome can serve as an indicator of the health of animals, and in the case of an ecosystem sentinel, like the SRW, may also indicate the health of the surrounding environment (Apprill 2017; Hazen et al. 2019).

Although previous approaches to understand SRW diet have helped address knowledge gaps, they each have their own biases, such as low taxonomic resolution and a reliance on taxonomic expertise and adequate reference databases. Molecular methods, such as faecal DNA metabarcoding, offer an efficient and non‐invasive approach that has now been widely adopted for diet studies (Deagle et al. 2007; O'Rorke et al. 2012; Ford et al. 2016). Metabarcoding has many advantages over conventional dietary methods such as morphological identification of prey remains from stomach contents (Lowry et al. 2023) or faeces (D'Agostino et al. 2016). It is swift, accurate to high taxonomic levels without the need for traditional taxonomic expertise, and capable of detecting trace amounts of highly digested prey DNA (Ando et al. 2020). A diet metabarcoding study of SRWs is yet to be performed and would offer timely insights to build on what is currently known of the species' diet.

This study aimed to characterise the diet and gut (faecal) microbiome of SRWs at different stages of their migratory cycle via DNA metabarcoding of faecal samples, and to examine associations between prey composition and gut bacterial composition. Henceforth, when used in relation to the samples in this study, the term gut microbiome refers to autochthonous and allochthonous bacteria detected in SRW faeces. To our knowledge, this is the first diet metabarcoding study to be performed on a right whale species and the first joint characterisation of whale diet and gut microbiome.

## 2 Methods

### 2.1 Sampling Locations

**FIGURE 1:**
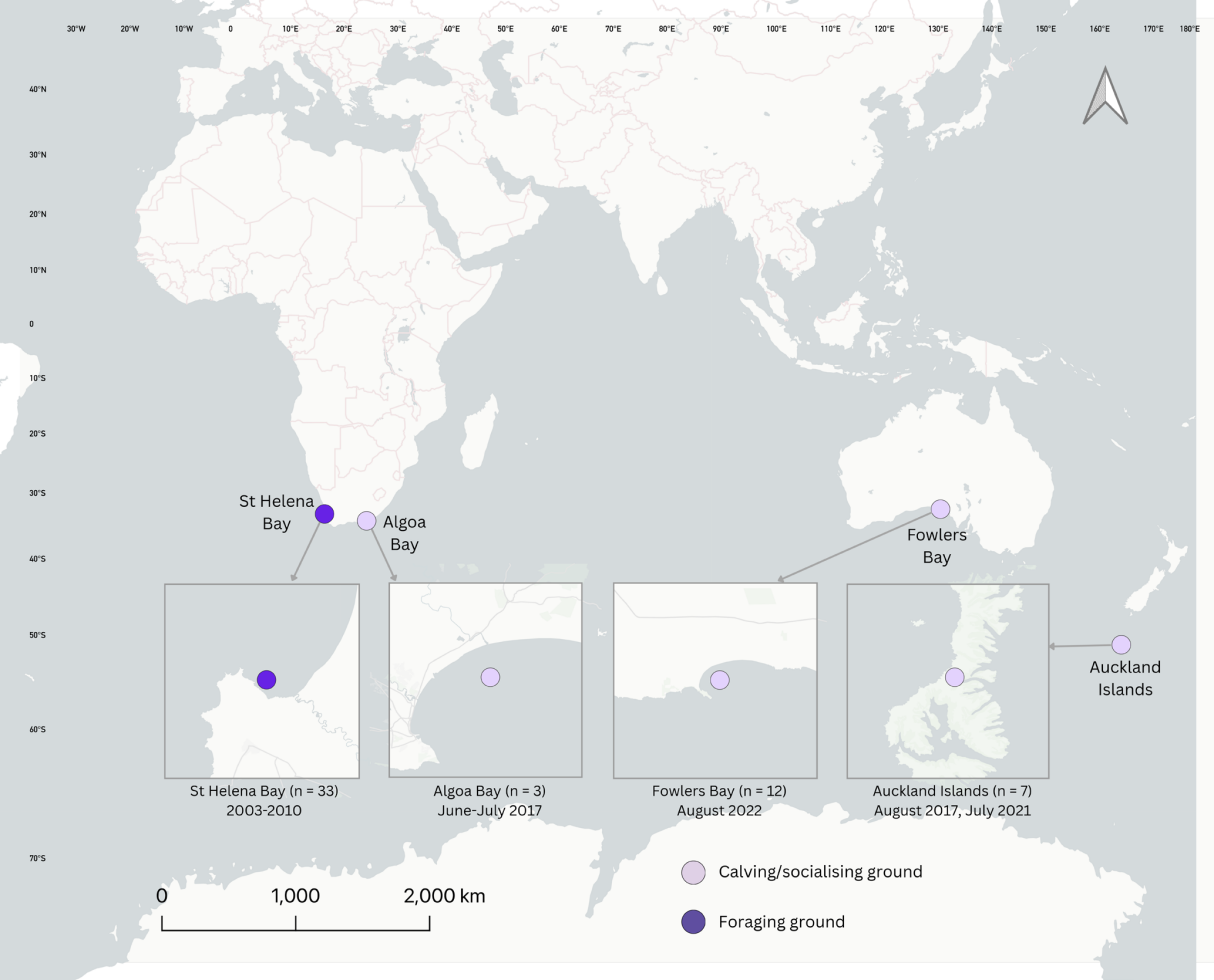
Map of sampling locations and periods for southern right whales from calving/socialising and foraging grounds, including number of samples from each location (*n*).

SRW faecal samples (*n* = 52) originated from the Auckland Islands, New Zealand; Fowlers Bay, Australia; Algoa Bay, South Africa; and St Helena Bay, South Africa (Figure 1). The Auckland Islands represent key calving and socialising grounds for the New Zealand SRW population (Carroll et al. 2022). All demographic classes of whales—cow‐calf pairs, adults and subadults—are present in high concentrations throughout the austral winter (Carroll et al. 2022; Derville et al. 2022), which is when samples for this study were collected (Figure 1). The waters surrounding the Auckland Islands support low‐biomass zooplankton communities dominated by copepods ( *Neocalanus tonsus* , *Calanus simillimus* , *Oithona similis* ), euphausiids (*Thysanoessa* spp.), salps, pteropods and chaetognaths (Bradford‐Grieve et al. 2003; Halfter et al. 2022). Fowlers Bay is a historically important Australian winter calving ground that SRWs have reoccupied in recent years, with peak relative abundance recorded in July and August, and a mean occupancy of 23 days for cow‐calf pairs compared with only 2 days for unaccompanied adults (Charlton et al. 2019; O'Shannessy et al. 2025). Samples for this study were collected in August 2022 (Figure 1). Fowlers Bay hosts a mesotrophic, copepod‐dominated zooplankton community structured by the Bonney Upwelling and supports the highest sardine and anchovy densities in Australian waters (van Ruth and Ward 2009; Kämpf et al. 2023). Algoa Bay offers warm waters with shallow reefs for SRWs to mate and calve during the austral winter, with SRWs present from June to November and samples for this study collected in June and July 2017 (Melly et al. 2018). In Algoa Bay, episodic upwelling and riverine input drive a seasonally influenced warm‐temperate zooplankton assemblage dominated by copepod species and meroplankton alongside pelagic fish larvae (Dali 2011; Costalago et al. 2018). Across these three locations, females consistently seek shallow, sheltered, nearshore habitat during the winter calving period, while males may spend more time socialising offshore (Best 1994). Additionally, calves were likely to be feeding/nursing, whereas adults were probably feeding only opportunistically to supplement their main diet.

In contrast, St. Helena Bay on the South African west coast functions primarily as a feeding and socialising area, with year‐round SRW presence recorded, and numbers peaking during the austral spring and summer (Best 1990; Barendse and Best 2014). A large proportion of the faecal samples analysed in this study were from St Helena Bay. Unfortunately, these were historical samples collected in past decades and stored for future work, and precise collection dates were not available for many. However, for those samples which did include a full date, collection took place during the austral spring and summer between the months of October and March. St. Helena Bay lies within the southern Benguela upwelling system and supports one of the region's most productive zooplankton communities, historically dominated by large calanoid copepods ( *Calanoides carinatus* , *Calanus australis*, *Centropages brachiatus*) and euphausiids ( *Euphausia lucens* ), though long‐term shifts towards smaller‐bodied species have been documented since the mid‐twentieth century (Gibbons and Hutchings 1996; Gibbons et al. 1999; Huggett et al. 2009). Across all four sampled systems, copepods appear to constitute the dominant zooplankton group.

### 2.2 DNA Extraction and Amplification

Samples were stored either frozen or suspended in 80%–90% ethanol (Table S1). All samples were extracted using a phosphate buffer extraction protocol (Taberlet et al. 2012) modified and optimised specifically for the purpose of targeting SRW prey (Parikh et al. 2026). In brief, approximately 200 mg of faecal material from each sample was incubated for 1 h in a saturated phosphate buffer and then centrifuged. The resulting pellet and supernatant were then extracted separately using the QIAGEN DNEasy PowerSoil kit (QIAGEN, Hilden, Germany). Extracts were quantified using a QuBit fluorometer (ThermoFisher Scientific, Massachusetts, USA), and pellet and supernatant extracts for each sample were combined in equimolar ratios prior to commencing PCRs. Whale diet was examined via two metabarcoding primers, an 18S rDNA eukaryote primer set and a 16S mtDNA crustacean‐specific primer, whereas gut microbiome was examined via a 16S rDNA bacterial primer (Table 1). PCRs were performed in triplicate for each sample within each primer set, and details of the PCR mixture and conditions for each may be found in Table S1. Sequencing for the two diet amplicons was performed on an Illumina NovaSeq X Plus sequencer (2 × 150 paired‐end), and bacterial sequencing was performed on an Illumina MiSeq Genome sequencer (2 × 250 bp paired‐end) at Auckland Genomics, University of Auckland—Waipapa Taumata Rau.

**TABLE 1:** Details of metabarcoding primers used in this study.

Primer name	Length (bp)	Target	Sequence	References
Crust16S_F(short)	170	16S mtDNA (Crustaceans)	GGG ACG ATA AGA CCC TAT A	Berry et al. (2017)
Crust16S_R(short)			ATT ACG CTG TTA TCC CTA AAG	Berry et al. (2017)
M13F_EukB	140–200	18S v9 rDNA (Eukaryotes)	TGT AAA ACG ACG GCC AGT TGA TCC TTC TGC AGG TTC ACC TAC	Medlin et al. (1988)
M13R_18s_v9_Con			CAG GAA ACA GCT ATG ACC CCT TTG TAC ACA CCG CCC	O'Rorke et al. (2012)
515F	390	16S v4 rDNA (Bacteria)	GTG **Y**CA GCM GCC GCG GTA A	Parada et al. (2016)
806R			GGA CTA C**N**V GGG TWT CTA AT	Apprill et al. (2015)

### 2.3 Bioinformatics Processing and Dataset Cleaning

Sequencing results for all three amplicons were processed using the Greenfield Hybrid Analysis Pipeline (GHAP; Greenfield 2017). Details of the bacterial 16S rDNA (hereafter referred to as 16S) bioinformatics and dataset cleaning are in Parikh et al. (2024). For the diet amplicons (Crust16S mtDNA and 18S rDNA, hereafter referred to as Crust16S and 18S, respectively), within GHAP, NCBI's BLAST tool was used to classify zero‐radius OTUs (zOTUs, i.e., 100% clustering). Demultiplexed reads were merged and trimmed to 80–120 bp (18S) and 130–180 bp (Crust16S) based on the majority of reads from histograms of read‐lengths. zOTU sequences were matched to NCBI's top 50 BLAST results from the GenBank database (Sayers et al. 2020) and aligned in MEGAN (Huson et al. 2007) for taxonomic classification. The zOTU tables for both diet datasets generated from the pipeline were then cleaned as follows: (1) for the 18S dataset, all non‐metazoan zOTUs or those unassigned to the phylum level were removed, and for the Crust16S dataset, any zOTUs not assigned to the subphylum Crustacea were removed; (2) the R package *decontam* was used to identify contaminant zOTUs (those with greater prevalence in negative controls than in true samples), and these zOTUs were removed (Davis et al. 2018); finally (3) any zOTUs with < 5 reads across all samples were removed (Toro‐Valdivieso et al. 2021). PCR replicates for each biological sample were combined by averaging their reads, as some replicates were lost during bioinformatics processing. zOTUs from duplicate species were also combined by averaging their reads. Read counts were converted to proportions (relative read abundance; RRA) within each sample for the results of each marker. The two markers did not pick up any overlapping species, genera, families or orders; and the respective datasets were combined by merging species proportions for each into one dataset. An RRA transformation was then performed on the combined dataset to normalise the two sets of results.

### 2.4 Statistical Analysis

Statistical analyses were performed in R (version 4.3.1; R Core Team 2023). Only samples in which prey were detected were considered for both diet and gut microbiome analysis, as the gut microbiome was being examined specifically for links to diet. Prey groups were summarised at the level of Order, and the frequency of detection (presence‐absence) of each prey group in whales from calving/socialising and foraging grounds was calculated. The frequency of detection of high‐abundance prey reads (> 0.15 RRA in a sample) was also calculated to counter for any bias towards less abundant groups. The RRA of prey groups in each sample was visualised via stacked bar plots using *ggplot2* (Wickham 2016). Gut bacterial classes with a total relative abundance > 0.05 were also plotted similarly.

Prey (species) and bacterial (zOTU) community data were both Hellinger transformed (square root of RRA) prior to the following analyses, which were carried out in base R and *vegan* (Oksanen et al. 2020), except where otherwise stated. To compare the composition of prey communities and bacterial communities between SRWs from calving/socialising and foraging grounds, a PERMANOVA was performed separately on each community dataset using the adonis2 function (Oksanen et al. 2020). A PERMANOVA was also carried out to test the effect of sampling location on prey and bacterial composition in whales from calving/socialising grounds. A Principal Component Analysis (PCA) was performed using the prcomp() function on each dataset to examine the variation in prey and bacterial communities. Prey and bacterial community data were subset for calving/socialising and foraging grounds, and a Procrustes test was performed on PCA ordinations for the two community datasets within each SRW group using the protest() function. Prey and bacterial community structures were also compared by generating Unweighted Pair Group Method with Arithmetic Mean (UPGMA) clustered dendograms via hclust() on Bray–Curtis dissimilarity matrices. The two dendograms were then combined into a tanglegram in *dendextend* (Galili 2015). For the following analyses, the RRA of prey groups was summarised at the level of Order and Hellinger transformed to use in place of species level prey community data. A redundancy analysis (RDA) was performed on bacterial communities (Hellinger transformed) with prey groups (Hellinger transformed) as explanatory variables. A stepwise model selection in both directions was performed on the RDA results using the ordistep() function with 1000 permutations to select prey groups responsible for the greatest variation, and ordination plots of bacterial communities with selected prey were created in *ggord* (Beck 2021).

## 3 Results

Raw sequencing results for 18S, Crust16S and 16S contained ~174 million, ~105 million and ~16 million paired‐end reads, respectively. The zOTU tables for each dataset before and after cleaning contained the following: for 18S, ~43 million reads and 839 zOTUs before cleaning and 13,912 reads and 43 zOTUs after, with an average of 266 (±147 SE) cleaned reads per sample; for Crust16S, ~90 million reads and 164 zOTUs before cleaning and ~11.7 million reads and 78 zOTUs after, with an average of 224,591 (±47,465 SE) reads per sample; and for 16S, ~8.3 million reads and 2368 zOTUs (97% clustering) before cleaning and ~1.8 million reads and 723 zOTUs after with an average of 34,920 (±1064 SE) reads per sample. After combining the two prey datasets (18S and Crust16S) for further analyses and merging duplicate species, the combined dataset contained 40 unique species/taxa.

### 3.1 Characterising the Prey and Gut Bacteria of SRWs From Calving/Socialising and Foraging Grounds

Putative prey were detected in 13 out of 22 (59%) calving/socialising ground samples and 26 out of 30 (86%) foraging ground samples. Crustaceans from the class Malacostraca dominated the overall prey detections (Table 2). In samples from calving/socialising grounds, prey detections were less frequent and generally lower in RRA compared to samples from the foraging ground.

**TABLE 2:** Frequency of detection of different prey groups (summarised at the order level) in samples from calving/socialising (*n* = 22) and foraging (*n* = 30) grounds. High RRA (relative read abundance) refers to the detection of prey groups with a read count > 15% of the total reads in a sample.

Marker	Phylum	Class	Order	Common name	Total detections		High RRA	
					Calving/socialising	Foraging	Calving/socialising	Foraging
18S	Acanthocephala	Palaeacanthocephala	Polymorphida	Parasitic worms	2	0	2	0
Crust16S	Arthropoda	Branchiopoda	Diplostraca	Cladocerans	6	10	5	4
18S	Arthropoda	Hexanauplia	Calanoida	Calanoid copepods	2	12	2	7
Crust16S	Arthropoda	Malacostraca	Amphipoda	Amphipods	0	4	0	1
Crust16S	Arthropoda	Malacostraca	Cumacea	Hooded shrimp	0	12	0	8
Crust16S	Arthropoda	Malacostraca	Decapoda	Crabs/lobsters/prawns	7	15	4	13
Crust16S	Arthropoda	Malacostraca	Euphausiacea	Krill	5	8	2	4
Crust16S	Arthropoda	Malacostraca	Isopoda	Isopods	1	0	1	0
Crust16S	Arthropoda	Malacostraca	Stomatopoda	Mantis shrimp	0	12	0	1
18S	Chaetognatha	Sagittoidea	Aphragmophora	Arrow worms	0	2	0	1
18S	Chordata	Ascidiacea	Phlebobranchia	Sea squirts	0	1	0	0
18S	Cnidaria	Hydrozoa	Leptothecata	Thecate hydroids	0	2	0	0
18S	Cnidaria	Scyphozoa	Scyphozoa	Jellyfish	0	3	0	1
18S	Cnidaria	Scyphozoa	Semaeostomeae	Jellyfish, other scyphozoans	0	11	0	6
18S	Mollusca	Bivalvia	Euheterodonta	Bivalves	1	0	0	0
18S	Mollusca	Bivalvia	Galeommatida	Saltwater clam	2	0	2	0
18S	Mollusca	Gastropoda	Nudibranchia	Nudibranchs	0	1	0	0
18S	Platyhelminthes	Trematoda	Plagiorchiida	Parasitic flatworms	1	0	1	0

#### 3.1.1 Prey Composition: Calving/Socialising Grounds

A total of 18 unique species were detected (Table S2). Decapoda (7 detections; 4 at high RRA, i.e., > 15% of the reads in a sample), Diplostraca (6; 5 high RRA) and Euphausiacea (5; 2 high RRA) were the main groups detected (Table 2). Calanoida were found in only two samples (Table 2). There were six decapod species, all crabs, with five of these detected at Fowlers Bay, two at Algoa Bay and one at the Auckland Islands (Table S2). The euphausiid species detected were *Nyctiphanes australis* (all three calving/socialising locations) and *Thysanoessa gregaria* (Fowlers Bay), and the only copepod identified below the Order level was *Parvocalanus crassirostris* (Algoa Bay; Table S2). The isopod *Scutuloidea maculata* was detected only at the Auckland Islands (Table S2). A few non‐crustacean taxa were unique to whales from calving/socialising grounds, including parasitic worms/flatworms (Plagiorchiida and Polymorphida, Auckland Islands) and bivalves (Euheterodonta and Galeommatida, Fowlers Bay; Table 2, Table S2).

#### 3.1.2 Prey Composition: Foraging Ground

**FIGURE 2:**
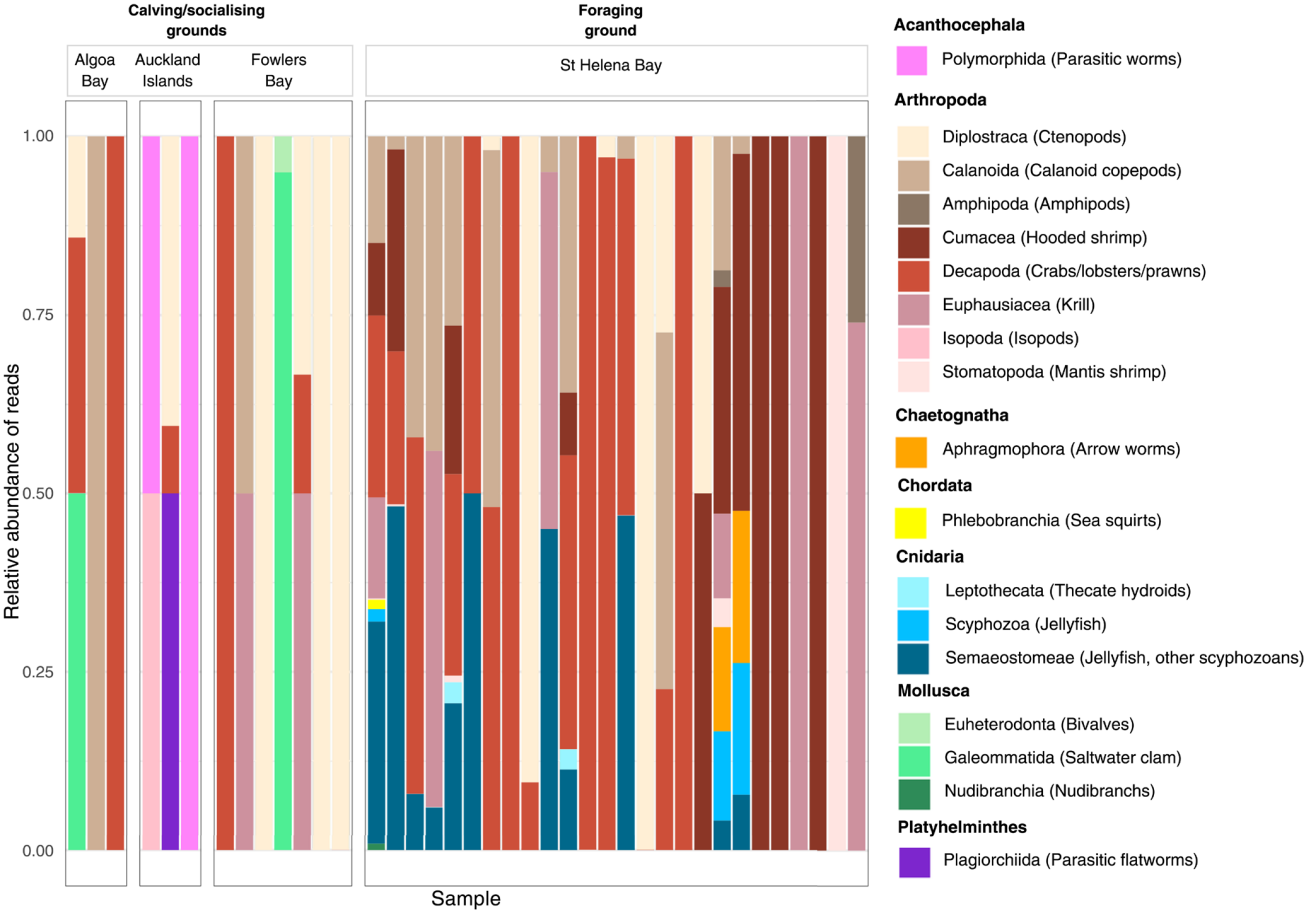
Proportion of prey groups (Orders) detected in whale faecal samples from calving/socialising and foraging grounds. Prey were detected in 59% of the samples from calving/socialising grounds and 86% of the samples from the foraging ground.

SRWs from the foraging ground showed a greater diversity and frequency of prey, with 29 unique species detected (Table S2). Decapoda were again the most frequently detected group (15 detections; 13 high RRA), spanning crab, lobster and prawn species (Figure 2; Table 2, Table S2). Cumacea (*Diastylis laevis*; 12 detections; 8 high RRA), Calanoida (12; 4 high RRA), Stomatopoda (*Pterygosquilla schizodontia*; 12; 1 high RRA) and Semaeostomeae (11; 6 high RRA) were detected frequently in whales from the foraging ground (Figure 2; Table 2, Table S2). Diplostraca ( *Penilia avirostris* ), Euphausiacea ( *Euphausia superba* and *T. gregaria* ) were detected in similar proportions to whales from calving/socialising grounds, but more frequently (Figure 2; Table 2, Table S2). A number of other groups were detected at low frequencies (Figure 2; Table 2). Overall, whales sampled at the foraging ground exhibited both higher prey diversity and more frequent high RRA detections than whales from calving/socialising grounds.

#### 3.1.3 Gut Microbiome Composition

Similar to prey, bacterial communities in whales from calving/socialising grounds were less diverse than those of whales from the foraging ground (Figure S1). Phylum *Firmicutes* dominated the gut microbiomes of whales from both calving/socialising and foraging grounds (mean relative abundance > 75% in both; Table S3). *Bacteroidetes* was the second most dominant phylum in whales from the foraging ground (12.6% ± 2.6 SE), demonstrating a much higher RRA compared to whales from calving/socialising grounds (1.3% ± 0.9; Table S3). *Actinobacteria* and *Proteobacteria* featured in the top five phyla in both groups of whales, while *Spirochaetota* was more common in whales from the foraging ground (3.6% ± 1.4; calving/socialising: 0.04% ± 0.02) and *Fusobacteria* was more prominent in whales from calving/socialising grounds (2.1% ± 2.1; foraging grounds: 0.01% ± 0.01; Table S3). At the level of class, *Clostridia* dominated in samples from calving/socialising grounds (63.8% ± 4.0) as well as the foraging ground (66.7 ± 3.8; Table S4). Whales from calving/socialising grounds then had notably high RRA of *Erysipelotrichia* (17.6% ± 4.7), while whales from the foraging ground had high proportions of *Bacteroidia* (12.5% ± 2.6; Table S4).

**FIGURE 3:**
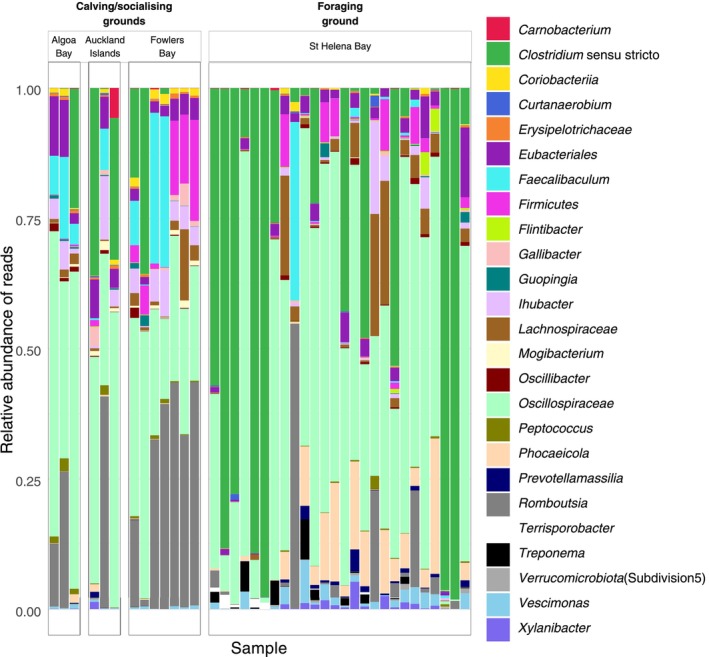
Proportion of top 25 most abundant bacterial genera (or higher taxonomic levels not resolved to the genus) detected in SRW faecal samples from both calving/socialising grounds and foraging grounds. Only samples where prey was also detected have been included (59% of the whales from calving/socialising grounds and 86% of the whales from the foraging grounds).

At a finer taxonomic scale, there were striking differences in the most proportionately abundant genera (Figure 3). *Romboutsia* (23.7% ± 5.5) followed by *Faecalibaculum* (16.1% ± 4.8) dominated the gut microbiomes of whales from calving/socialising grounds (Table S5). *Clostridium* sensu stricto dominated the foraging ground samples (28.7% ± 6.3), though also present in relatively high proportions within calving/socialising grounds samples (8.0% ± 3.5; Table S5). *Phocaeicola* was proportionately the second most abundant genus in foraging ground samples (7.7% ± 1.7), present in much higher RRA compared to calving/socialising grounds (0.4% ± 0.2), and *Vescimonas* also featured more prominently in whales from the foraging ground (3.6 ± 0.9) compared to calving/socialising grounds (0.6% ± 0.1; Table S5).

### 3.2 Comparing the Composition of Prey and Gut Bacterial Communities in SRWs From Calving/Socialising and Foraging Grounds

Multivariate analyses revealed significant differences between whales from calving/socialising and foraging grounds in both prey and gut bacterial communities, albeit the magnitude of separation was stronger for bacteria. Prey communities (Hellinger transformed) differed significantly between migratory stages (PERMANOVA: *F* = 3.4; *p* = 0.002). The PCA ordination showed some clustering of calving/socialising grounds samples, while foraging ground samples were more spread out, and PCA axes 1 and 2 explained 32% of the variation (Figure S2a). Gut bacterial communities (also Hellinger transformed) showed an even clearer differentiation based on migratory stage (PERMANOVA: *F* = 7.2; *p* = 0.001), with a clearer division in clustering between calving/socialising and foraging grounds, and PCA axes 1 and 2 capturing 46.9% of the variation (Figure S2b).

Within calving/socialising grounds, prey communities did not differ significantly among locations (PERMANOVA: *F* = 1.3; *p* = 0.210), the sample sizes likely being too low to detect any pattern. Gut bacterial communities showed moderately significant variation among calving/socialising locations (PERMANOVA: *F* = 2.1; *p* = 0.030), with pairwise tests showing that whales from Fowlers Bay harboured distinct microbiomes compared to those at Algoa Bay (*p* = 0.029) and the Auckland Islands (*p* = 0.048).

The relationship between prey and bacterial community composition was significant at both SRW migratory stages, but stronger in whales from calving/socialising grounds (Procrustes analysis: *m* ^2^ = 0.34, correlation = 0.81, *p* = 0.010), where nearly two‐thirds of the bacterial variation (66%) was explained by prey composition, as compared to the foraging ground (*m* ^2^ = 0.49, correlation = 0.71, *p* = 0.001), where just over half of the variation (51%) was explained. It should be noted again that the low sample sizes from calving grounds make it challenging to detect significant patterns, as can be seen from the higher significance of results from the foraging ground. The tanglegram also supported the association, with samples clustered similarly across prey and bacterial communities at both migratory stages, suggesting that individuals with comparable prey profiles tended to host similar gut bacterial assemblages (Figure S3).

**FIGURE 4:**
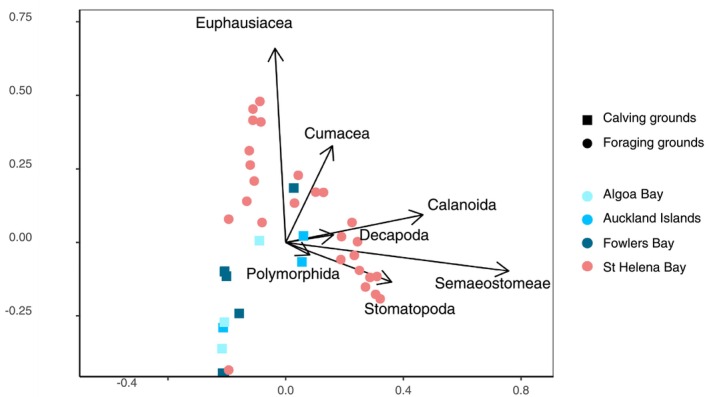
RDA of bacterial communities of whales from calving/socialising and foraging grounds with prey groups selected via stepwise selection as explanatory variables.

The role of SRW prey as predictors of gut bacterial communities was examined via an RDA ordination. Prey groups, summarised at the level of Order, collectively explained 53% of the variation in gut bacterial communities. Stepwise model selection highlighted a subset of prey groups—Cumacea, Decapoda, Euphausiacea, Calanoida, Polymorphida, Semaeostomeae and Stomatopoda—as the strongest predictors of bacterial community variation, jointly explaining 37% of the total variation (Figure 4). Whales from calving/socialising and foraging grounds clustered differently, with the parasitic worms Polymorphida being the only group associated with the former (Figure 4). Euphausiacea and Semaeostomeae were the strongest drivers of bacterial community structure (Figure 4). Whales from the foraging ground grouped in two clusters, with Euphausiacea driving one group and the remaining prey driving the other (Figure 4). All the selected prey groups except Cumacea and Decapoda tested significantly in an ANOVA of the stepwise model results (Table S6).

## 4 Discussion

Diet studies on free‐ranging whales are challenging due to their wide‐ranging habitats across remote oceanic areas and therefore the opportunistic nature of obtaining samples (Reidy et al. 2022). Here, we present a unique dataset which examines both the diet and gut microbiome of SRWs across different migratory stages and populations. We found that SRWs, previously reported to have a diet dominated by euphausiids and copepods (Tormosov et al. 1998; D'Agostino et al. 2024), consumed a diverse range of prey at the low latitude foraging ground of this study, spanning crustaceans, jellyfish, molluscs and other occasional taxa. Importantly, our results showed that Decapoda (crabs/lobsters/prawns) may form a key component of the SRW diet across all the sampled locations, alongside Calanoida (copepods), Stomatopoda (mantis shrimp), Cumacea (hooded shrimp) and Semaeostomeae (jellyfish) at the sampled foraging ground. We also show that diet plays a significant role in shaping the SRW gut microbiome, with Euphausiacea and Semaeostomeae having the strongest influence on bacterial community structure.

### 4.1 New Insights Into SRW Diet at Calving/Socialising and Foraging Grounds

SRW feeding at calving/socialising grounds has been explored mainly around Península Valdés, Argentina, where calanoid copepods are the dominant prey, although various other taxa have also been found associated with the SRW diet (Hoffmeyer et al. 2010; D'Agostino et al. 2016, 2018, 2023, 2024). In our study, we detected decapods, cladocerans and euphausiids at all the calving/socialising locations and copepods at Fowlers Bay and Algoa Bay, but not at the Auckland Islands. Zooplankton sampling at Península Valdés has found Decapoda to be a dominant mesozooplankton group in areas where SRWs feed (D'Agostino et al. 2018, 2023). Squat lobsters ( *G. gregaria* ) are decapods, and SRWs have been observed feeding on these (Matthews 1938; D'Agostino et al. 2018; Carroll unpublished data). However, ours is the first study to identify this group as potentially a key component of SRW diet, being detected more frequently and in higher proportions than either euphausiids or copepods at SRW calving/socialising grounds as well as at the St Helena Bay foraging ground. Cladocerans ( *P. avirostris* ) were similarly detected at high frequency and proportions in both calving/socialising and foraging grounds, and have also been detected in SRW feeding patches in Península Valdés (D'Agostino et al. 2018). In the eastern Great Australian Bight, encompassing Fowlers Bay, the Bonney Upwelling drives seasonal mesozooplankton increases, which would extend to decapod larvae and cladocerans (van Ruth and Ward 2009). SRWs have already been observed feeding on decapods ( *G. gregaria* ) near the Auckland Islands (Carroll unpublished data), and decapods and other crustaceans are known to occur in east South African waters (Singh et al. 2021). Therefore, it is not surprising that SRWs at these sites may encounter decapods and cladocerans during episodes of locally dense meroplanktonic aggregation.

The patterns of euphausiids and copepods detected at the calving/socialising grounds were partially consistent with Soviet records indicating that copepods were the main dietary component of SRWs north of 40° S and euphausiids south of 50° S between 1951 and 1971 (Tormosov et al. 1998). Algoa Bay and Fowlers Bay are both above 40° S, while the Auckland Islands are around 50° S. *Nyctiphanes australis* (Algoa Bay, Auckland Islands, Fowlers Bay) and *Thysanoessa gregaria* (Fowlers Bay) were the euphausiids consumed at calving/socialising grounds. *N. australis* is common around New Zealand and Australia (Bradford and Chapman 1988; O'Brien 1988) and its sister species *N. capensis* is found at Algoa Bay (Cornew et al. 1992); whereas *T. gregaria* is primarily distributed between 30° S and 50° S (Kulagin et al. 2024). The detection of these species appears to reflect regional taxonomic patterns, but it is noteworthy that euphausiids were more dominant than copepods at these locations. The latter were detected in only two samples from calving/socialising grounds—one from Algoa Bay and one from Fowlers Bay, although this may also be a reflection of low sample sizes, particularly at Algoa Bay (*n* = 3). Overall, the prey detections here suggest that Fowlers Bay and Algoa Bay are calving/socialising grounds that support supplementary feeding in SRWs to a greater extent than the Auckland Islands, where less prey were detected.

At the St Helena Bay foraging ground, we found that Decapoda, Calanoida, Cumacea, Stomatopoda and Semaeostomeae were the most frequently detected prey groups. Copepods are numerically the most dominant zooplankton group in the southern Benguela region which encapsulates St Helena Bay (Gibbons and Hutchings 1996; Huggett et al. 2009). Recent work along the South African west coast (St Helena Bay to Cape Point) found that copepods dominated zooplankton samples near foraging SRWs as well as faecal prey remains from three SRWs (Van Zyl 2023). The prominence of Decapoda in our dietary detections therefore does not reflect bulk zooplankton community composition or expected SRW prey in the region. However, upwelling systems like the southern Benguela are characterised by strong spatiotemporal fluctuations in zooplankton communities (Gibbons et al. 1999). In fact, non‐copepod zooplankton communities within St Helena Bay are known to change significantly within the span of days, driven by the dynamics of both their biological and physical environments (Gibbons et al. 1999). Cumaceans (*Diastylis* spp.) and brachyuran decapod larvae have been documented as components of the non‐copepod zooplankton in St Helena Bay, with their abundance subject to episodic, physically‐driven variables (e.g., low oxygen) rather than sustained numerical dominance (Gibbons et al. 1999). Thus, SRWs may encounter these prey during periods of locally elevated abundance, consistent with the opportunistic foraging strategy proposed by Kenney (2009), that is, non‐selectively foraging on densely aggregated available prey. Scyphozoan jellyfish such as the order Semaeostomeae may similarly occur as episodic blooms in St Helena Bay and the broader southern Benguela, driven by interannual variability in temperature and oceanographic conditions (Buecher and Gibbons 2000). In contrast to metabarcoding, jellyfish and other soft‐bodied organisms would not be detected by traditional prey remains analyses (Carroll et al. 2019; Bucklin et al. 2024). SRWs at Peninsula Valdés have been observed repeatedly feeding on other gelatinous zooplankton (mainly ctenophores) in spring (D'Agostino et al. 2023). It is therefore plausible that gelatinous zooplankton may be a targeted prey choice for SRWs, particularly during favourable seasons. However, it must be noted that they are relatively low in lipid content, which is an essential nutritional component of the SRW diet (Hsieh and Rudloe 1994; Harcourt et al. 2019).

#### 4.1.1 Challenges and Considerations When Interpreting Diet Results

The less frequent, lower RRA prey detections at calving/socialising grounds compared to foraging grounds could be attributed to two causes, one being that whales from calving/socialising grounds were feeding opportunistically and less frequently, and the second being that a number of these samples originated from calves whose diet is dominated by milk. Samples from Fowlers Bay, which constituted 54% of the total calving/foraging grounds samples, were thought to originate largely from calves, although specific information for each sample was not available (R. Keogh, pers comm). However, the age class for the remaining samples was unknown. While SRW calves have been observed feeding on zooplankton alongside mothers' milk at Península Valdés, further study is required to understand the extent and role of supplementary feeding in calves (D'Agostino et al. 2023). Therefore, it must be considered that the calving/socialising grounds of this study may have included a significant proportion of calves, and direct comparisons of the amount/diversity of prey consumed may not be representative of fully foraging adults.

Although DNA metabarcoding has vastly enhanced the way we study animal diets, it is not without limitations. The relative abundance of prey items obtained via metabarcoding is not always a reliable representation of actual prey consumption, however, neglecting abundance can lead to overrepresentation of negligible prey items (Deagle et al. 2019). For this reason, we used a mix of relative abundance and frequency of detection. The dominant presence of Decapoda in terms of both frequency and relative abundance must be interpreted with some caution. A 2021 metabarcoding study of South African zooplankton found that DNA records of regionally occurring crustacean species were sorely underrepresented in the BOLD reference database (Singh et al. 2021). Only 6% of crustaceans known to occur in South Africa had barcodes available, with Leptostraca (50% of 4 species), Mysida (21% of 58 species) and Decapoda (10% of 750 species) being best represented. Our study used the GenBank reference database, however, similar biases towards the dominance of commercially important regional species may be true here as well. We know there is also a risk of false positives with eDNA metabarcoding, particularly in baleen whales, when non‐prey items may be incidentally ingested during filter feeding (Reidy et al. 2022), or from faecal sample contamination from the surrounding water/air (Sepulveda et al. 2020). Primer bias may also lead to false negatives (Serrana et al. 2019). For example, we found that each diet primer (18S rDNA and Crust16S mtDNA) captured unique compositions, even at the level of Order, emphasising the need for metabarcoding studies to employ a multi‐locus approach (Taberlet et al. 2018; Carroll et al. 2019).

The relatively lower detection of Calanoida (copepods) in this study compared to Decapoda also warrants consideration, given their established importance in the diet of SRWs and their numerical dominance in the zooplankton communities of the study regions (Tormosov et al. 1998; van den Berg et al. 2021). Copepod detections were exclusively derived from the 18S rDNA marker, which yielded substantially lower read depth after bioinformatics cleaning than the Crust16S marker (mean 266 ± 147 vs. 224,591 ± 47,465 reads per sample). This disparity in sensitivity may have contributed to a slight under‐detection of copepod DNA and reinforces the potential impacts of primer bias. Additionally, copepods lack the calcium storage mechanism common to most other crustacean groups, leaving them with a comparatively thin, weakly sclerotised exoskeleton (Luquet 2012). This softer exoskeleton relative to the other chitinous crustaceans detected by the Crust16S marker may have resulted in more rapid digestion and DNA degradation prior to defecation, further reducing their RRA (Luquet 2012; Snider et al. 2021).

Prior to this study, SRW diet has been studied via stomach content analysis (Tormosov et al. 1998), visual observation (Hamner et al. 1988), zooplankton sampling, video recordings (D'Agostino et al. 2024), stable isotope analysis (van den Berg et al. 2021) and faecal analysis of prey remains (D'Agostino et al. 2016). Diet metabarcoding studies on other species have also revealed broader diversities of prey than traditional methods (Bucklin et al. 2024). Overall, our findings, which suggest a frequent consumption of decapods, stomatopods, cumaceans and jellyfish alongside the expected prey of copepods, point to a capacity to adjust to other regional prey in the face of changing krill availability (Agrelo et al. 2021).

### 4.2 Relationship Between Prey and Gut Microbiome

Our study is the first to characterise the prey and gut microbial composition of a baleen whale. Associations between the two have been observed in terrestrial animals (Li et al. 2022). Our results showed distinct gut microbial communities in whales from calving/socialising and foraging grounds, with significant associations to prey communities at both migratory stages, and with Euphausiacea the strongest driver of bacterial variation. Traditionally, *E. superba* were expected to dominate the SRW diet only below 50°S (Tormosov et al. 1998), but this study points to a more complex picture, with *E. superba* detected at a low‐latitude foraging ground and *N. australis* and *T. gregaria* at mid‐latitude calving grounds. Although these euphausiids were not detected at great frequency or relative abundance in our samples, their close association to the gut microbiome could point towards their long‐term role as a dominant SRW prey (Tormosov et al. 1998). Diet and gut microbiome are, after all, temporally discrete. While prey composition derived from a faecal sample is a snapshot into what the host has most recently consumed (Franz et al. 2023), the gut microbiome reflects a stable core composition over a long period of time, shaped by phylogeny as well as diet (Erwin et al. 2017). In addition to euphausiids and copepods, a number of diverse prey taxa were selected as the key predictors of bacterial communities, suggesting that diet–microbiome interactions in SRWs are not only driven by crustaceans, but also by other prey groups consumed during foraging (e.g., jellyfish) as well as non‐prey taxa (e.g., parasites) encountered during calving, which may introduce unique microbial signatures.

The most notable difference between the microbiomes of whales from calving/socialising and foraging grounds was the increased proportion of *Bacteroidetes* in whales from the foraging ground. *Bacteroidetes* are known for their role in digesting complex polysaccharides into short‐chain fatty acids (SCFAs) butyrate, acetate and propionate, as well as for metabolising micronutrients, such as iron and folate (Tanca et al. 2017). SRWs sampled from the foraging ground likely contain higher relative abundances of *Bacteroidia* to meet their increased need to digest chitin‐rich prey (complex polysaccharides). Indeed, the most dominant genus in whales from the foraging ground was *Clostridium*, which, although belonging to *Firmicutes*, is known for its production of chitinases (Dierick et al. 2024). Whales at calving/socialising grounds were distinguished by the presence of *Erysipelotrichia* in higher proportions compared to whales from the foraging ground. *Erysipelotrichia* is associated with lipid metabolism, essential for whales which rely on blubber reserves (Kaakoush 2015). Further, the genera *Romboutsia* and *Faecalibaculum* were enriched in whales from calving/socialising grounds, both of which have been associated with mammalian milk and meconium (Ge et al. 2021; Naspolini et al. 2025). The majority of samples collected at Fowlers Bay were presumed to have been from calves (R. Keogh, pers comm); however, the age class of whales at Algoa Bay and the Auckland Islands was not known. In particular, the enrichment of *Romboutsia* in most of the Fowlers Bay samples suggests its potential as a microbial signature for calves.

## 5 Conclusion

SRW feeding in mid‐latitude foraging grounds has remained stable since the late 1700s, while high‐latitude foraging grounds have been less frequented in recent decades (Derville et al. 2022). Our findings reveal more generalist feeding behaviour than previously suggested for the species at a low‐latitude foraging ground as well as supplementary feeding at calving/socialising grounds, potentially by calves as well as adults. Given the observed northward shift in foraging (van den Berg et al. 2021; Derville et al. 2022), understanding what prey resources are responsible for supporting populations at lower latitudes is essential for continued recovery of the species and to further piece together the complex interplay between health, nutritional stress, reproduction and population growth (Seyboth et al. 2016; Charlton et al. 2022; Vermeulen et al. 2023). Declines in SRW maternal body condition (Vermeulen et al. 2023), and with it, calving rates (Charlton et al. 2022; Vermeulen et al. 2025), have been linked to the availability of prey at high latitudes (Seyboth et al. 2016; van den Berg et al. 2021). The emergence of Decapoda, Stomatopoda, Cumacea and even Semaeostomeae as potentially important prey groups for SRWs at low latitudes calls for the remodelling of climate change scenarios and deeper investigation into the role of these prey groups in SRW foraging and survival. Are they consumed widely across populations and latitudes, and are they capable of supporting population growth? In the context of changing ecosystems under the impact of climate change, shifts in prey availability could also alter gut microbiome composition over a period of time, with the interplay of the two jointly affecting host health (Moloney et al. 2014). Establishing an association between diet and the allochthonous and autochthonous gut microbiome and monitoring their relationship can act as an early warning for changes to the species and the ecosystem (Apprill 2017).

## Author Contributions

A.P. wrote the original draft; A.P., E.L.C., E.V., R.H., R.O. and A.C. contributed to project design; E.L.C., E.V., S.P. and W.R. provided whale faecal samples; A.P. conducted statistical analyses; R.O., E.L.C., E.V., R.H. and A.C. supervised the project, and all authors reviewed and edited the manuscript.

## Funding

Open Access funding was enabled and organised by CAUL and its Member Institutions. The Collection of SRW faecal samples from the Maungahuka Auckland Islands, New Zealand was supported by: The Royal Society—Te Apārangi Rutherford Discovery Fellowship, Live Ocean, Lou and Iris Fisher Charitable Trust, Joyce Fisher Charitable Trust, Brian Sheth/Sangreal Foundation, University of Auckland (U Auckland) Science Faculty Research Development Fund, International Whaling Commission—Southern Ocean Research Partnership, Antarctic and Southern Ocean Coalition, New Zealand Department of Conservation—Te Papa Atawhai and the Cawthron Institute. Collection of SRW faecal samples from Algoa Bay, South Africa was funded by Transnet National Ports Authority (TNPA), South Africa; the Collaborative Postgraduate Training Programme at Higher Education Institutions (HEI) in partnership with other universities, industry and government (Grant ID: 92525), National Research Foundation (NRF), South Africa; and Technology and Human Resources for Industry Programme (THRIP, Grant ID: 90207) funding from the National Research Foundation (NRF), South Africa.

## Disclosure

Benefit sharing: Benefits from this research include the sharing of results from preliminary stages and lay summaries at acceptance with local stakeholders (EP Cruises, Indigenous groups) and the public sharing of data as outlined above.

## Conflicts of Interest

The authors declare no conflicts of interest.

## Supporting information


**Figure S1:** Proportion of gut bacterial classes (or higher taxonomic levels not resolved to class) with > 5% total relative abundance, detected in whale faecal samples from calving/socialising and foraging grounds. Only samples where prey was also detected have been included (59% of the whales from calving/socialising grounds and 86% of the whales from the foraging grounds).
**Figure S2:** Principal Component Analysis (PCA) of Hellinger transformed (a) SRW prey communities and (b) gut bacterial communities, with prey communities grouped by SRW migratory stages: calving/socialising and foraging.
**Figure S3:** Tanglegram comparing the clustering of gut bacterial and prey communities in the sampled SRWs. The straight bars connecting the prey and bacterial communities suggest high similarity in community structure, with significant clusters highlighted in dark pink. Sample names are coloured in light purple for calving/socialising SRWs and dark purple for foraging SRWs.
**Table S1:** PCR conditions for the three metabarcoding primers used in this study. PCRs were performed in two rounds with conditions for the second round being the same for all three primer sets.
**Table S2:** Frequency of prey at finest taxonomic resolution detected at each calving/socialising ground (Algoa Bay, Auckland Islands and Fowlers Bay) and foraging ground (St Helena Bay).
**Table S3:** Relative abundances of bacterial phyla detected in the gut microbiomes of SRWs from calving/socialising and foraging grounds.
**Table S4:** Relative abundances of top 10 most abundant bacterial classes (or higher taxonomic levels not resolved to the genus) detected in the gut microbiomes of SRWs from calving/socialising and foraging grounds each.
**Table S5:** Relative abundances of top 25 most abundant bacterial genera (or higher taxonomic levels not resolved to the genus) detected in the gut microbiomes of SRWs from calving/socialising and foraging grounds each.
**Table S6:** ANOVA results for stepwise selection model used to select SRW prey groups significantly associated with gut bacterial communities.

## Data Availability

Raw sequencing data for this project are available on NCBI SRA under accession number PRJNA1285085 (https://www.ncbi.nlm.nih.gov/sra/PRJNA1285085). All other data associated with this study including bioinformatics results, Supporting Information, metadata and R code are publicly available on *Dryad* (https://doi.org/10.5061/dryad.83bk3jb6f).
