# Supplementary material for: DNA Metabarcoding Reveals Unexpected Predator–Prey–Microbial Dynamics in the Southern Right Whale (Eubalaena australis)
Source: Mol Ecol. 2026 Jun 15;35(12):e70442. doi: 10.1111/mec.70442 (PMC13266376; doi:10.1111/mec.70442)
Supplement: Supplementary file 1 — Figure S1: Proportion of gut bacterial classes (or higher taxonomic levels not resolved to class) with > 5% total relative abundance, detected in whale faecal samples from calving/socialising and foraging grounds. Only samples where prey was also detected have been included (59% of the whales from calving/socialising grounds and 86% of the whales from the foraging grounds). Figure S2: Principal Component Analysis (PCA) of Hellinger transformed (a) SRW prey communities and (b) gut bacterial communities, with prey communities grouped by SRW migratory stages: calving/socialising and foraging. Figure S3: Tanglegram comparing the clustering of gut bacterial and prey communities in the sampled SRWs. The straight bars connecting the prey and bacterial communities suggest high similarity in community structure, with significant clusters highlighted in dark pink. Sample names are coloured in light purple for calving/socialising SRWs and dark purple for foraging SRWs. Table S1: PCR conditions for the three metabarcoding primers used in this study. PCRs were performed in two rounds with conditions for the second round being the same for all three primer sets. Table S2: Frequency of prey at finest taxonomic resolution detected at each calving/socialising ground (Algoa Bay, Auckland Islands and Fowlers Bay) and foraging ground (St Helena Bay). Table S3: Relative abundances of bacterial phyla detected in the gut microbiomes of SRWs from calving/socialising and foraging grounds. Table S4: Relative abundances of top 10 most abundant bacterial classes (or higher taxonomic levels not resolved to the genus) detected in the gut microbiomes of SRWs from calving/socialising and foraging grounds each. Table S5: Relative abundances of top 25 most abundant bacterial genera (or higher taxonomic levels not resolved to the genus) detected in the gut microbiomes of SRWs from calving/socialising and foraging grounds each. Table S6: ANOVA results for stepwise selection model used to select SRW pr [file MEC-35-e70442-s001.docx]

**DNA metabarcoding reveals unexpected predator-prey-microbial dynamics in the southern right whale (*Eubalaena australis*)**

*Southern right whale diet and gut microbiome*

Aashi Parikh^a^*, Richard O’Rorke^b^, Emma L. Carroll^b^, Els Vermeulen^c^, Robert Harcourt^a^, Stephanie Plön^d^, William J. Rayment^e^, Anthony Chariton^a^

^a^School of Natural Sciences, Macquarie University, Wallumattagal Campus, Macquarie Park NSW 2109 Australia ([aashi-chetan.parikh@hdr.mq.edu.au](mailto:aashi-chetan.parikh@hdr.mq.edu.au); [robert.harcourt@mq.edu.au](mailto:robert.harcourt@mq.edu.au); [anthony.chariton@mq.edu.au](mailto:anthony.chariton@mq.edu.au))

^b^School of Biological Sciences, Waipapa Taumata Rau – University of Auckland, Auckland 1142 Aotearoa - New Zealand ([roro002@aucklanduni.ac.nz](mailto:roro002@aucklanduni.ac.nz); [e.carroll@auckland.ac.nz](mailto:e.carroll@auckland.ac.nz))

^c^Mammal Research Institute Whale Unit, Faculty of Natural and Agricultural Sciences, University of Pretoria, Hatfield 0028 South Africa ([els.vermeulen@up.ac.za](mailto:els.vermeulen@up.ac.za))

^d^BioConsult SH, Schobüller Str. 36, Husum 25813 Germany ([s.ploen@bioconsult-sh.de](mailto:s.ploen@bioconsult-sh.de))

^e^Department of Marine Science, Ōtākou Whakaihu Waka - University of Otago, Dunedin 9016 Aotearoa – New Zealand ([will.rayment@otago.ac.nz](mailto:will.rayment@otago.ac.nz))

*Corresponding author – [aashi-chetan.parikh@hdr.mq.edu.au](mailto:aashi-chetan.parikh@hdr.mq.edu.au)

**Table S1.** PCR conditions for the three metabarcoding primers used in this study. PCRs were performed in two rounds with conditions for the second round being the same for all three primer sets.

| **PCR Round 1** | | | | | | |  |
| --- | --- | --- | --- | --- | --- | --- | --- |
| **Amplicon** | **PCR Mixture** | | **PCR Conditions** | | | |  |
| **18S** | **Reagent** | **Volume (uL)** | **PCR Stage** | **Temperature** | **Time** | **PCR cycles** |  |
|  | Mastermix | 10 | Initial denaturation | 95°C | 8 min |  |  |
|  | Primer-F (0.5uM) | 3.5 | Denaturation | 95°C | 20 s | x 35 |  |
|  | Primer-R (0.5uM) | 3.5 | Annealing | 58°C | 20 s |  |  |
|  | PNA clamp* | 1 | Elongation | 72°C | 30 s |  |  |
|  | DNA | 2 | Final elongation | 72°C | 8 min |  |  |
| **Crust16S** | Mastermix | 10 | Initial denaturation | 95°C | 10 min |  |  |
|  | Primer-F (0.5uM) | 4 | Denaturation | 95°C | 20 s | x 45 |  |
|  | Primer-R (0.5uM) | 4 | Annealing | 51°C | 30 s |  |  |
|  | DNA | 2 | Elongation | 72°C | 45 s |  |  |
|  |  |  | Final elongation | 72°C | 10 min |  |  |
| **16S** | Mastermix | 10 | Initial denaturation | 95°C | 10 min |  |  |
|  | Primer-F (0.5uM) | 4 | Denaturation | 95°C | 20 s | x 30 |  |
|  | Primer-R (0.5uM) | 4 | Annealing | 50°C | 30 s |  |  |
|  | DNA | 2 | Elongation | 72°C | 45 s |  |  |
|  |  |  | Final elongation | 72°C | 5 min |  |  |
| **PCR Round 2** | | | | | | |  |
| The second round of PCR for all 3 amplicons added Illumina Unique Dual Indexes (UDIs) from index adapter sets A, B and C. Each PCR reaction was 16 mL, including Q5 High-Fidelity 2X Master Mix (New England Biolabs, Ipswich, MA, USA), 0.2 mM UDI, and 1 mL of product from the first round PCR diluted to 5%. The PCR conditions were: 30s of initial denaturation at 98°C; 14x PCR cycles of 10s denaturation at 98°C, 10s annealing at 57°C and 20s elongation at 72°C; concluding with a 1 min final elongation at 72°C.  *Whale blocking PNA clamp: CGACCGTCTTCTCAGC-Lys | | | | | | |  |
|  |  |  |  |  |  |  |  |
|  |  |  |  |  |  |  |  |
|  |  |  |  |  |  |  |  |
|  |  |  |  |  |  |  |  |
|  |  |  |  |  |  |  |  |

**Table S2.** Frequency of prey at finest taxonomic resolution detected at each calving/socialising ground (Algoa Bay, Auckland Islands and Fowlers Bay) and foraging ground (St Helena Bay).

| **Order** | **Species** | **Algoa Bay** | **Auckland Islands** | **Fowlers Bay** | **St Helena Bay** |
| --- | --- | --- | --- | --- | --- |
| Polymorphida | Polymorphidae | 0 | 2 | 0 | 0 |
| Diplostraca | *Penilia avirostris* | 1 | 1 | 4 | 10 |
| Calanoida | Calanoida | 0 | 0 | 1 | 9 |
| Calanoida | *Parvocalanus crassirostris* | 1 | 0 | 0 | 0 |
| Neocopepoda | Neocopepoda | 1 | 0 | 1 | 10 |
| Amphipoda | *Cyamus boopis* | 0 | 0 | 0 | 4 |
| Cumacea | *Diastylis laevis* | 0 | 0 | 0 | 12 |
| Decapoda | *Epilobocera capolongoi* | 0 | 0 | 2 | 0 |
| Decapoda | *Goneplax rhomboides* | 0 | 0 | 0 | 1 |
| Decapoda | *Hymenosoma geometricum* | 0 | 0 | 0 | 1 |
| Decapoda | Hymenosoma orbiculare | 0 | 0 | 0 | 2 |
| Decapoda | Jasus | 0 | 0 | 0 | 1 |
| Decapoda | Monodaeus | 0 | 0 | 0 | 5 |
| Decapoda | *Mursia cristiata* | 0 | 0 | 0 | 4 |
| Decapoda | *Neoxanthops quadrilobatus* | 0 | 0 | 1 | 0 |
| Decapoda | *Ovalipes trimaculatus* | 0 | 0 | 0 | 4 |
| Decapoda | Penaeoidea | 0 | 0 | 0 | 1 |
| Decapoda | *Pilumnoides perlatus* | 0 | 0 | 2 | 11 |
| Decapoda | Pilumnus | 0 | 0 | 0 | 0 |
| Decapoda | *Plagusia chabrus* | 1 | 0 | 0 | 1 |
| Decapoda | Portunus | 1 | 0 | 2 | 4 |
| Decapoda | *Thia scutellata* | 0 | 1 | 1 | 5 |
| Decapoda | Xanthidae | 1 | 0 | 0 | 0 |
| Euphausiacea | Euphausia | 0 | 0 | 0 | 5 |
| Euphausiacea | *Euphausia superba* | 0 | 0 | 0 | 2 |
| Euphausiacea | *Nyctiphanes australis* | 1 | 1 | 2 | 0 |
| Euphausiacea | *Thysanoessa gregaria* | 0 | 0 | 1 | 2 |
| Euphausiacea | *Thysanoessa* sp. BD-2006 | 0 | 0 | 0 | 3 |
| Isopoda | *Scutuloidea maculata* | 0 | 1 | 0 | 0 |
| Stomatopoda | *Pterygosquilla schizodontia* | 0 | 0 | 0 | 12 |
| Aphragmophora | Sagittidae | 0 | 0 | 0 | 2 |
| Phlebobranchia | *Ciona intestinalis* | 0 | 0 | 0 | 1 |
| Leptothecata | Leptothecata | 0 | 0 | 0 | 2 |
| Scyphozoa | Scyphozoa | 0 | 0 | 0 | 3 |
| Semaeostomeae | Chrysaora | 0 | 0 | 0 | 11 |
| Semaeostomeae | Pelagiidae | 0 | 0 | 0 | 2 |
| Euheterodonta | Euheterodonta | 0 | 0 | 1 | 0 |
| Galeommatida | Montacutidae | 1 | 0 | 1 | 0 |
| Nudibranchia | *Jorunna tomentosa* | 0 | 0 | 0 | 1 |
| Plagiorchiida | Plagiorchiida | 0 | 1 | 0 | 0 |

**Table S3.** Relative abundances of bacterial phyla detected in the gut microbiomes of SRWs from calving/socialising and foraging grounds.

| **Calving/socialising** | | | **Foraging** | | |
| --- | --- | --- | --- | --- | --- |
| **Phylum** | **Mean relative abundance (%)** | **SE** | **Phylum** | **Mean relative abundance (%)** | **SE** |
| *Firmicutes* | 87.06 | 2.27 | *Firmicutes* | 76.22 | 3.20 |
| *Actinobacteria* | 8.38 | 1.09 | *Bacteroidetes* | 12.59 | 2.62 |
| *Fusobacteria* | 2.13 | 2.08 | *Actinobacteria* | 5.04 | 1.06 |
| *Bacteroidetes* | 1.33 | 0.92 | *Spirochaetota* | 3.58 | 1.36 |
| *Proteobacteria* | 0.48 | 0.33 | *Proteobacteria* | 1.05 | 0.26 |
| *Verrucomicrobiota* | 0.43 | 0.16 | *Verrucomicrobiota* | 1.02 | 0.26 |
| *Synergistota* | 0.09 | 0.05 | *Euryarchaeota* | 0.19 | 0.06 |
| *Spirochaetota* | 0.04 | 0.02 | *Mycoplasmatota* | 0.10 | 0.04 |
| *Euryarchaeota* | 0.04 | 0.03 | *Synergistota* | 0.10 | 0.03 |
| *Lentisphaerota* | 0.02 | 0.01 | *Lentisphaerota* | 0.09 | 0.04 |
| *Mycoplasmatota* | 0.001 | 0.001 | *Fusobacteria* | 0.01 | 0.01 |
| *Cyanobacteriota* | 0.000 | 0.000 | *Cyanobacteriota* | 0.004 | 0.002 |
| *Planctomycetota* | 0.000 | 0.000 | *Planctomycetota* | 0.003 | 0.002 |

**Table S4.** Relative abundances of top 10 most abundant bacterial classes (or higher taxonomic levels not resolved to the genus) detected in the gut microbiomes of SRWs from calving/socialising and foraging grounds each.

| **Calving/socialising** | | | **Foraging** | | |
| --- | --- | --- | --- | --- | --- |
| **Order** | **Mean relative abundance (%)** | **SE** | **Order** | **Mean relative abundance (%)** | **SE** |
| *Clostridia* | 63.82 | 4.01 | *Clostridia* | 66.68 | 3.79 |
| *Erysipelotrichia* | 17.61 | 4.68 | *Bacteroidia* | 12.53 | 2.62 |
| *Coriobacteriia* | 7.48 | 1.03 | *Erysipelotrichia* | 6.52 | 2.20 |
| *Firmicutes*_c | 3.00 | 1.13 | *Coriobacteriia* | 3.78 | 0.77 |
| *Bacilli* | 2.49 | 2.31 | *Spirochaetia* | 3.58 | 1.36 |
| *Fusobacteriia* | 2.13 | 2.08 | *Firmicutes*_c | 1.92 | 0.63 |
| *Bacteroidia* | 1.31 | 0.91 | *Actinobacteria*_c | 1.25 | 0.65 |
| *Actinobacteria*_c | 0.90 | 0.12 | *Bacilli* | 0.63 | 0.30 |
| *Gammaproteobacteria* | 0.43 | 0.31 | *Verrucomicrobiota* | 0.60 | 0.20 |
| *Verrucomicrobiota*_c | 0.29 | 0.13 | *Betaproteobacteria* | 0.49 | 0.18 |

**Table S5.** Relative abundances of top 25 most abundant bacterial genera (or higher taxonomic levels not resolved to the genus) detected in the gut microbiomes of SRWs from calving/socialising and foraging grounds each.

| **Calving/socialising** | | | **Foraging** | | |
| --- | --- | --- | --- | --- | --- |
| **Genus** | **Mean relative abundance (%)** | **SE** | **Genus** | **Mean relative abundance (%)** | **SE** |
| *Romboutsia* | 23.69 | 5.45 | *Clostridium sensu stricto* | 28.68 | 6.34 |
| *Faecalibaculum* | 16.12 | 4.79 | *Phocaeicola* | 7.68 | 1.72 |
| *Clostridium sensu stricto* | 8.02 | 3.47 | *Oscillospiraceae*_g | 7.65 | 1.31 |
| *Coriobacteriia*_g | 5.23 | 0.80 | *Romboutsia* | 4.61 | 1.77 |
| *Ihubacter* | 4.47 | 0.69 | *Vescimonas* | 3.63 | 0.92 |
| *Oscillospiraceae*_g | 4.23 | 0.91 | *Treponema* | 3.55 | 1.35 |
| *Peptococcus* | 3.96 | 1.02 | *Terrisporobacter* | 3.48 | 1.36 |
| *Firmicutes*_g | 3.00 | 1.13 | *Faecalibaculum* | 2.80 | 1.68 |
| *Mediterraneibacter* | 2.71 | 0.77 | *Faecalitalea* | 2.75 | 1.58 |
| *Guopingia* | 2.51 | 1.47 | *Flintibacter* | 2.60 | 1.28 |
| *Carnobacterium* | 2.30 | 2.15 | *Lachnospiraceae*_g | 2.46 | 0.81 |
| *Cetobacterium* | 2.13 | 2.08 | *Prevotellamassilia* | 2.16 | 0.63 |
| *Mogibacterium* | 2.12 | 0.46 | *Firmicutes*_g | 1.92 | 0.63 |
| *Peptacetobacter* | 1.95 | 0.76 | *Ihubacter* | 1.78 | 0.58 |
| *Gallibacter* | 1.66 | 0.86 | *Coriobacteriia*_g | 1.58 | 0.56 |
| *Dorea* | 1.21 | 0.37 | *Peptococcus* | 1.58 | 0.35 |
| *Eubacteriaceae*_g | 1.17 | 0.14 | *Guopingia* | 1.49 | 0.88 |
| *Lachnospiraceae*_g | 1.03 | 0.47 | *Xylanibacter* | 1.22 | 0.37 |
| *Eubacteriales*_g | 0.94 | 0.21 | *Curtanaerobium* | 1.08 | 0.40 |
| *Erysipelotrichaceae*_g | 0.88 | 0.15 | *Bariatricus* | 0.99 | 0.43 |
| *Atopobiaceae*_g | 0.81 | 0.40 | *Eubacteriales*_g | 0.93 | 0.19 |
| *Oscillibacter* | 0.81 | 0.28 | *Bifidobacterium* | 0.89 | 0.62 |
| *Lentihominibacter* | 0.61 | 0.15 | *Oscillibacter* | 0.88 | 0.18 |
| *Vescimonas* | 0.55 | 0.13 | *Mediterraneibacter* | 0.85 | 0.46 |
| *Curtanaerobium* | 0.50 | 0.15 | *Dorea* | 0.70 | 0.21 |

**Fig. S1**. Proportion of gut bacterial classes (or higher taxonomic levels not resolved to class) with >5% total relative abundance, detected in whale faecal samples from calving/socialising and foraging grounds. Only samples where prey was also detected have been included (59% of the whales from calving/socialising grounds and 86% of the whales from the foraging grounds).


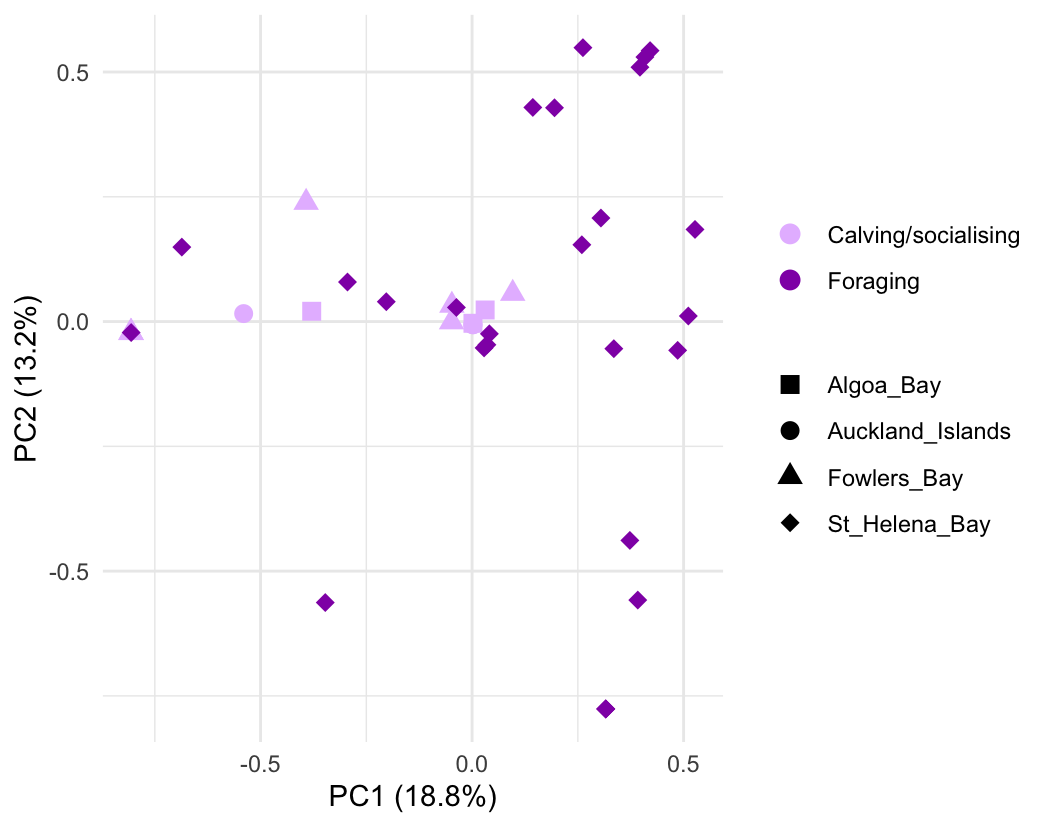

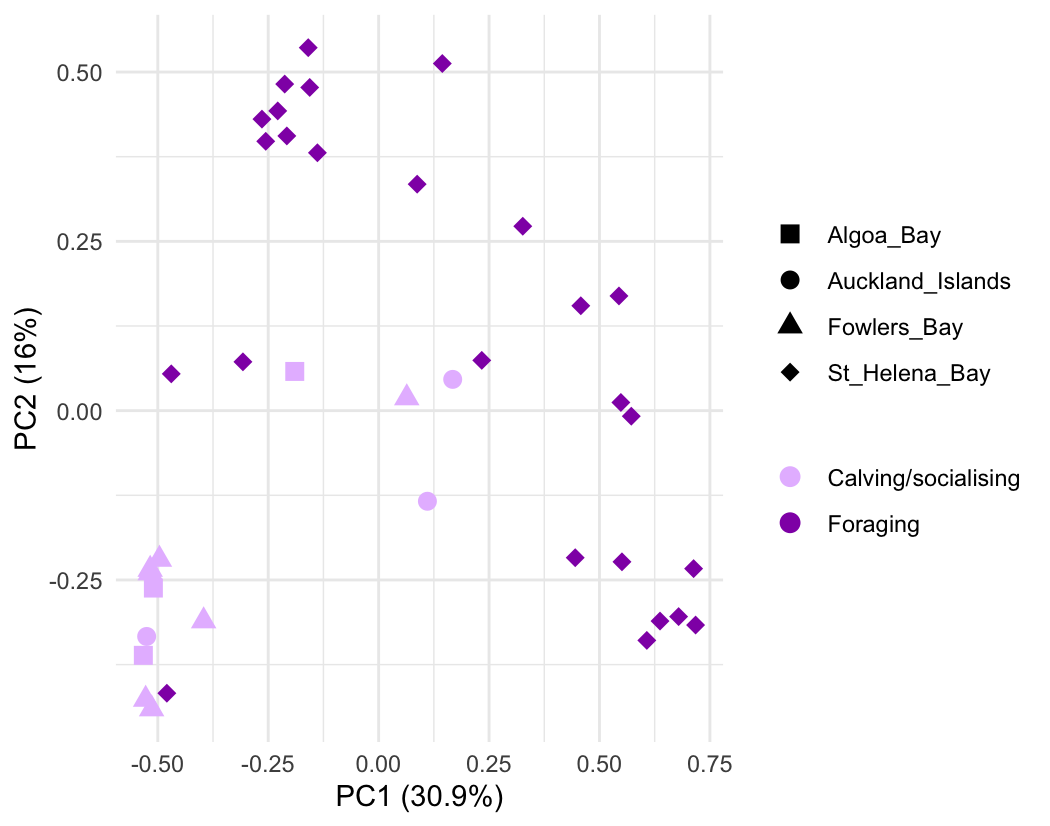


**Fig. S2**. Principal Component Analysis (PCA) of Hellinger transformed (a) SRW prey communities and (b) gut bacterial communities, with prey communities grouped by SRW migratory stages: calving/socialising and foraging

**
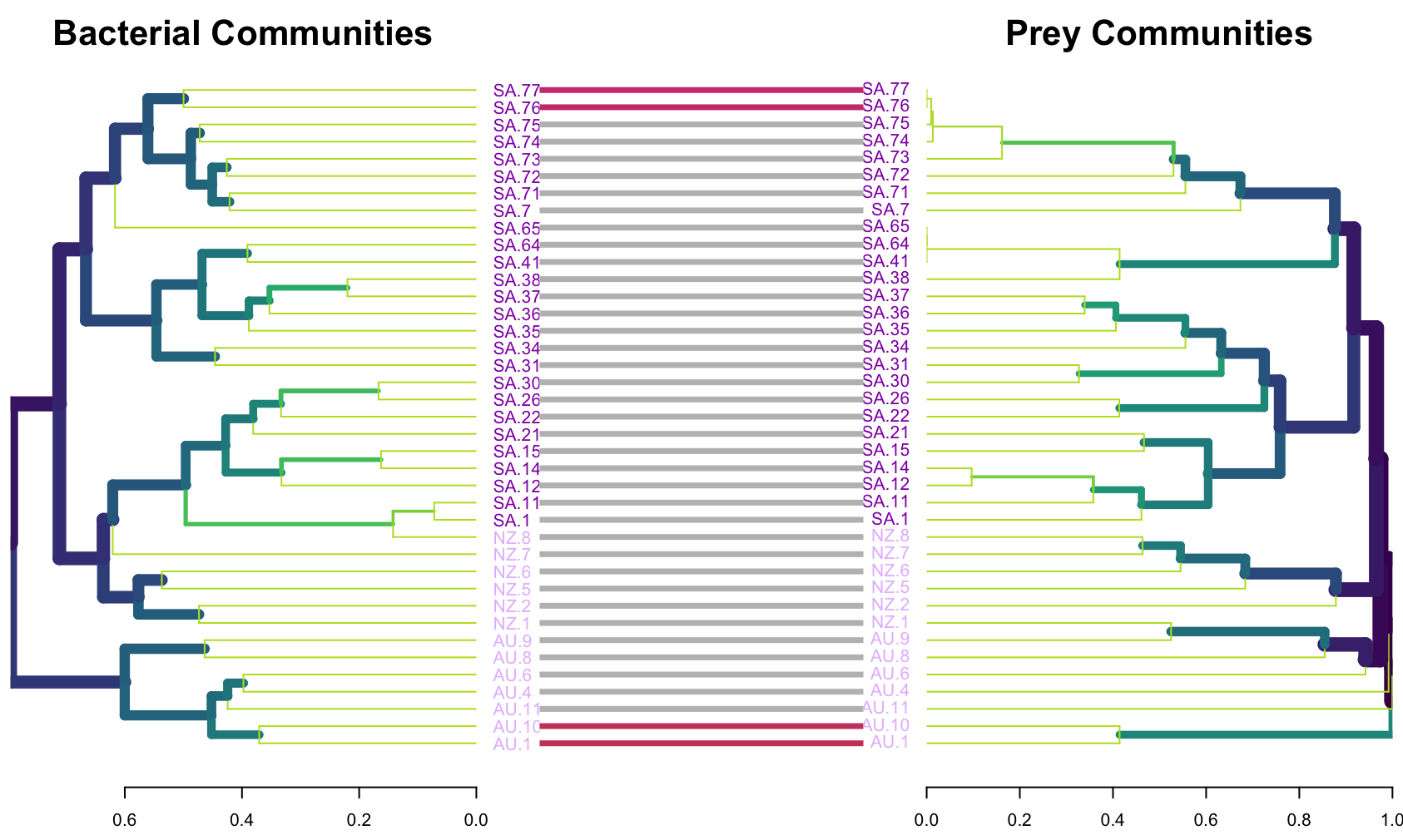
**

**Fig. S3.** Tanglegram comparing the clustering of gut bacterial and prey communities in the sampled SRWs. The straight bars connecting the prey and bacterial communities suggest high similarity in community structure, with significant clusters highlighted in dark pink. Sample names are coloured in light purple for calving/socialising SRWs and dark purple for foraging SRWs.

**Table S6.** ANOVA results for stepwise selection model used to select SRW prey groups significantly associated with gut bacterial communities.

|  | Df | Variance | F | Pr(>F) |  |
| --- | --- | --- | --- | --- | --- |
| Cumacea | 1 | 0.01556 | 1.2212 | 0.203 |  |
| Decapoda | 1 | 0.01693 | 1.329 | 0.19 |  |
| Euphausiacea | 1 | 0.02977 | 2.3364 | 0.025 | * |
| Calanoida | 1 | 0.03685 | 2.8919 | 0.009 | ** |
| Polymorphida | 1 | 0.02604 | 2.0437 | 0.022 | * |
| Semaeostomeae | 1 | 0.07392 | 5.8009 | 0.001 | *** |
| Stomatopoda | 1 | 0.03317 | 2.6027 | 0.001 | *** |
